# Plasma Oxylipins: A Potential Risk Assessment Tool in Atherosclerotic Coronary Artery Disease

**DOI:** 10.3389/fcvm.2021.645786

**Published:** 2021-04-21

**Authors:** D. Elizabeth Le, Manuel García-Jaramillo, Gerd Bobe, Armando Alcazar Magana, Ashish Vaswani, Jessica Minnier, Donald B. Jump, Diana Rinkevich, Nabil J. Alkayed, Claudia S. Maier, Sanjiv Kaul

**Affiliations:** ^1^Knight Cardiovascular Institute, Oregon Health and Science University, Portland, OR, United States; ^2^Nutrition Program, School of Biological and Population Health Sciences, Oregon State University, Corvallis, OR, United States; ^3^Linus Pauling Institute, Oregon State University, Corvallis, OR, United States; ^4^Helfgott Research Institute, National University of Natural Medicine, Portland, OR, United States; ^5^Department of Animal and Rangeland Sciences, Oregon State University, Corvallis, OR, United States; ^6^Department of Chemistry, Oregon State University, Corvallis, OR, United States; ^7^Department of Biostatistics and Knight Cancer Institute, Oregon Health and Science University, Portland, OR, United States; ^8^Department of Anesthesiology and Perioperative Medicine, Oregon Health and Science University, Portland, OR, United States

**Keywords:** coronary artery disease, oxylipins, diagnosis, prognosis, mass spectrometry, LCMS

## Abstract

**Background:** While oxylipins have been linked to coronary artery disease (CAD), little is known about their diagnostic and prognostic potential.

**Objective:** We tested whether plasma concentration of specific oxylipins may discriminate among number of diseased coronary arteries and predict median 5-year outcomes in symptomatic adults.

**Methods:** Using a combination of high-performance liquid chromatography (HPLC) and quantitative tandem mass spectrometry, we conducted a targeted analysis of 39 oxylipins in plasma samples of 23 asymptomatic adults with low CAD risk and 74 symptomatic adults (≥70% stenosis), aged 38–87 from the Greater Portland, Oregon area. Concentrations of 22 oxylipins were above the lower limit of quantification in >98% of adults and were compared, individually and in groups based on precursors and biosynthetic pathways, in symptomatic adults to number of diseased coronary arteries [(1) *n* = 31; (2) *n* = 23; (3) *n* = 20], and outcomes during a median 5-year follow-up (no surgery: *n* = 7; coronary stent placement: *n* = 24; coronary artery bypass graft surgery: *n* = 26; death: *n* = 7).

**Results:** Plasma levels of six quantified oxylipins decreased with the number of diseased arteries; a panel of five oxylipins diagnosed three diseased arteries with 100% sensitivity and 70% specificity. Concentrations of five oxylipins were lower and one oxylipin was higher with survival; a panel of two oxylipins predicted survival during follow-up with 86% sensitivity and 91% specificity.

**Conclusions:** Quantification of plasma oxylipins may assist in CAD diagnosis and prognosis in combination with standard risk assessment tools.

## Introduction

Coronary artery disease (CAD) is the leading cause of death worldwide (1–3). Traditional CAD risk factors such as diabetes, smoking, hypertension, hyperlipidemia, and family history of premature cardiovascular disease (CVD), as well as nontraditional risk factors of rheumatic inflammatory disease, human immunodeficiency disease, and gestational diabetes can assist clinicians in decisions for CAD primary prevention but have limited efficacy in high CAD risk patient management (4). This is currently done using expensive invasive tests, such as exercise stress testing (without or with concomitant imaging for myocardial perfusion and/or function) or measuring the coronary calcium score on X-ray computed tomography (CT). Our objective is to develop a point-of-care blood test that could assist in decision making regarding CAD patient management.

Ruptured arterial plaques are a major reason for adverse cardiac events with resultant thrombus formation that partially or completely impairs blood flow to the heart (5). The progression from asymptomatic to ruptured arterial plaques involves lipid oxidation and inflammation (6, 7). Conventional indicators of lipid oxidation include secondary products such as 4-hydroxynonenal (4-HNE) (8), malondialdehyde (MDA) (9), and oxidized low-density lipoproteins (Ox-LDL) (10), and its associated oxidized phospholipids (11). Technological advancements in soft ionization tandem mass spectrometry (MS/MS) have allowed multiplexed quantification of oxidized lipids in one analytical run and with it the emergence of isoprostanes and oxylipins as indicators of oxidative tissue injuries, implicating oxidative tissue injuries in the pathology of a variety of chronic diseases (3, 12).

**Figure 1:**
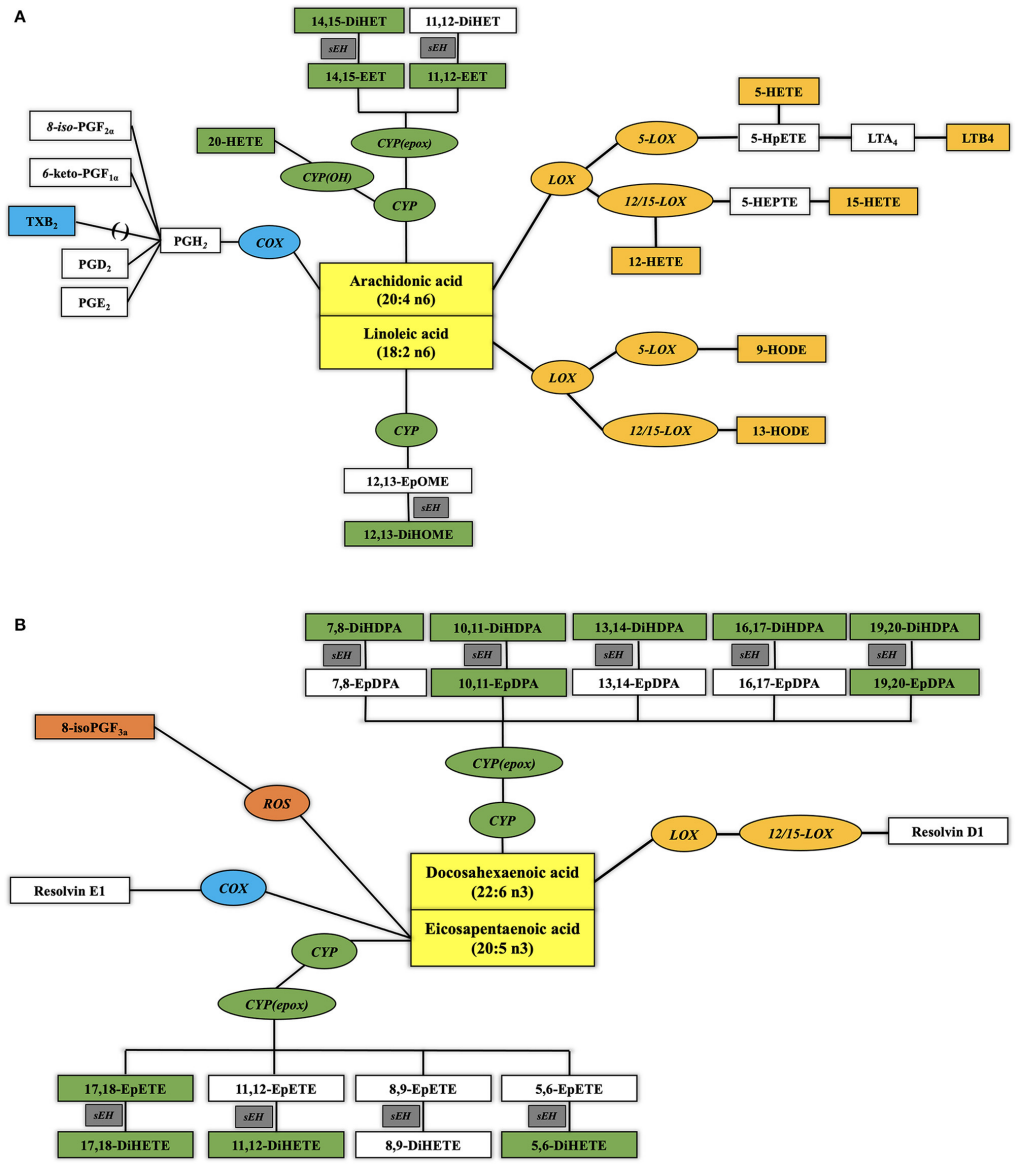
Biosynthetic pathways of plasma oxylipins from omega-6 **(A)** and omega-3 **(B)** polyunsaturated fatty acids (PUFA). Normal text designates PUFA (yellow rectangular boxes) and oxylipins. Italic text designates enzymes involved in the metabolic transformation [blue oval boxes for cyclo-oxygenases or aspirin, orange oval boxes for lipoxygenases or CYP1B1, green oval boxes for cytochrome P450, and gray rectangular boxes for soluble epoxide hydrolase (sEH)] with their quantified oxylipins in the same color and oxylipins below the limit of quantification (LOQ) in noncolored squares. See Abbreviation Index and Appendix for full names description.

Oxylipins are oxidized long and very long chain polyunsaturated fatty acids (PUFA), which are derived from phospholipids. Oxylipins can be classified based on their fatty acid (FA) precursor (Figure 1). The dominant precursors of oxylipins are the more proinflammatory omega-6 PUFA linoleic acid (LA; C18:2 *n*−6) and arachidonic acid (ARA; C20:4 *n*−6) and the more anti-inflammatory omega-3 PUFA linolenic acid (LNA; C18:3 *n*−3), eicosapentaenoic acid (EPA; C20:5 *n*−3), and docosahexaenoic acid (DHA; C22:6 *n*−3). The dominant oxylipin biosynthesis pathways are named after the enzymes involved, as follows: lipoxygenases (LOX), cyclooxygenases (COX), cytochrome P450 (CYP450) epoxygenases and hydroxylases, and soluble epoxide hydrolases. Reactive oxygen species (ROS) can initiate the formation of a few, specific oxylipins from PUFAs (13). Prior human studies with limited sample sizes reported elevated concentrations of ARA-derived oxylipins in unstable arterial plaques (14) and ischemic heart tissue (15). Elevated circulating concentrations were observed in individuals after cardiac surgery (16) and those experiencing adverse cardiac events on follow-up (17, 18). Furthermore, CAD patients had higher circulating concentrations of ARA-derived oxylipins than non-CAD adults (19–21).

In the present study, we evaluated whether plasma oxylipins, alone or in panels, may discriminate among number of diseased coronary arteries and predict median 5-year outcomes in high CAD risk symptomatic patients and ≥70% stenosis which, to our knowledge, has not been previously published. In doing so, a point-of-care plasma oxylipin test could assist in decision making regarding CAD patient management. Here, we report results of a small study confirming this hypothesis.

## Materials and Methods

### Participants and Study Design

**Figure 2:**
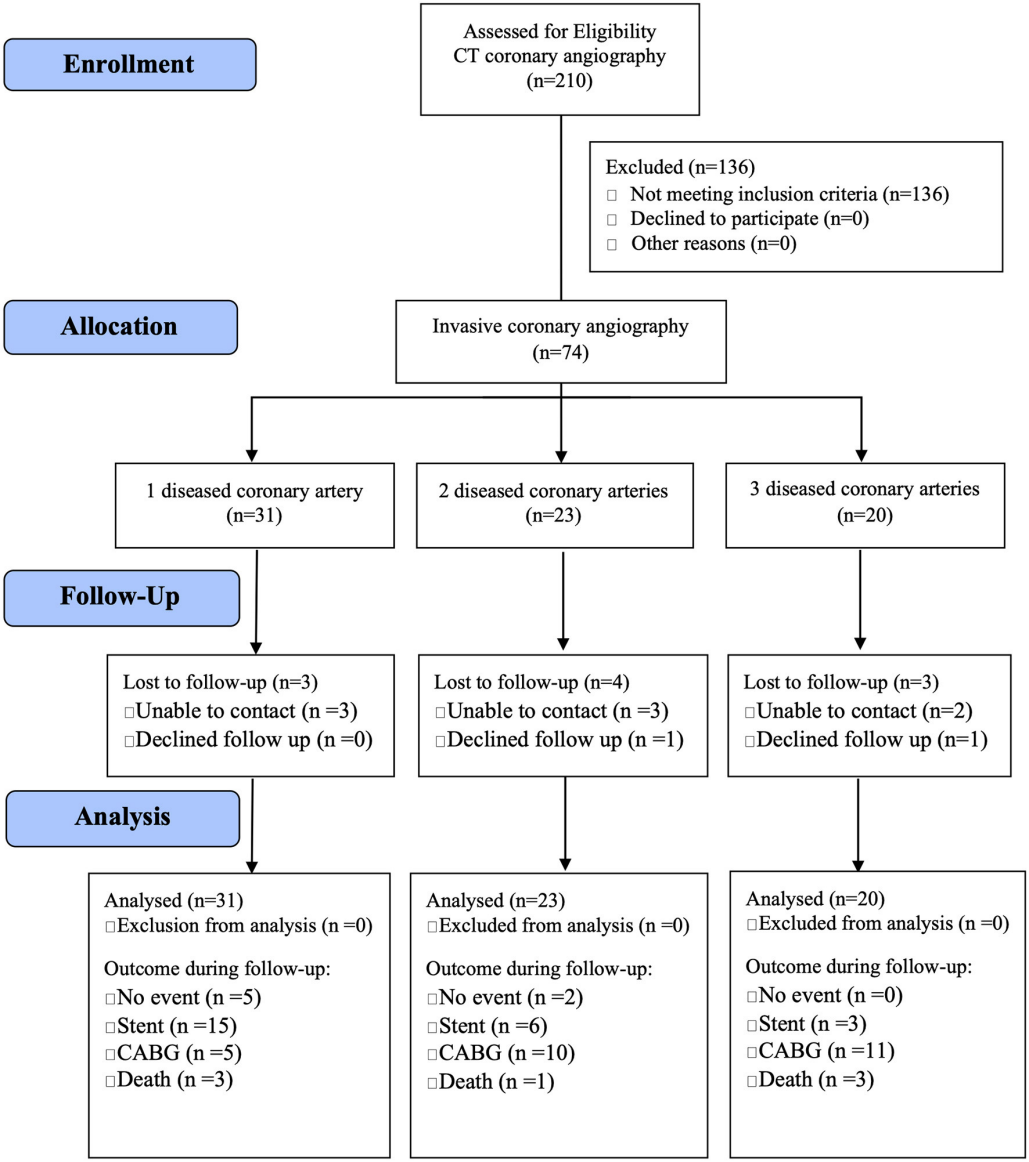
Study flow diagram of adults with diseased coronary arteries (≥70% stenosis) from the greater Portland area, Oregon.

The study was approved by the Institutional Review Board of the Oregon Health and Science University (OHSU) in Portland, Oregon. We prospectively enrolled 74 individuals from the greater Portland metropolitan area from October 2012 and January 2017 (IRB00008606) who were referred to OHSU for an invasive coronary angiography because of symptoms suggestive of CAD (median age: 66 years; range: 38–87 years). Inclusion criteria were inducible myocardial ischemia during stress (either on echocardiography or single-photon computed tomography) and ≥70% coronary luminal narrowing of one or more major coronary artery or its major branch on subsequent coronary angiography. Exclusion criteria were <70% coronary stenosis on angiography, prior myocardial infarction, hemodynamically significant valvular heart disease, prior revascularization, or congestive heart failure. The CAD patients were classified as having one-vessel (*n* = 31), two-vessel (*n* = 23), or three-vessel (*n* = 20) CAD and were followed up until November 2019 (Figure 2) for a median of 60 months (range: 25–84 months) for adverse events [i.e., coronary stent placement; coronary artery bypass graft (CABG) surgery; death]. Ten CAD patients were lost to follow-up (unable to contact: *n* = 8; declined to follow-up: *n* = 2).

To establish ranges of plasma oxylipin concentrations in low CAD risk populations, we established the Astoria cohort and prospectively enrolled 220 individuals from Astoria, a rural community within the same area as the CAD patients, from July 2016 to February 2017 (IRB00011193). For the current study, we selected individuals of the same age range (range: 38–71 years) that fulfilled all the exclusion criteria (*n* = 23). Exclusion criteria were self-reported history of hyperlipidemia, diabetes, myocardial infarction, ischemia, coronary artery revascularization surgery, coronary atherosclerosis on coronary angiography, active tobacco use, and family history of CAD. Participants from the Astoria and Portland were not age-matched.

### Sample Collection and Preparation for Oxylipin Analysis

All participants fasted for at least 6 h before 4.5 mL blood was collected in tubes containing 0.01 M buffered sodium citrate and immediately placed on ice. Blood samples were collected 1–4 h prior to coronary angiography of participants with CAD. Whole blood samples were then centrifuged at 3,000 rpm for 15 min in a refrigerated centrifuge at 4°C, after which the plasma was aliquoted into 1 mL Eppendorf tubes and immediately stored at −80°C until analysis.

Oxylipins from plasma were extracted as described in Pedersen et al. (22) with minor modifications. The internal oxylipin standards used during the extraction (Supplementary Table 1) were used to correct the recovery of the quantified oxylipins (23).

### Chromatographic and Mass Spectrometric Analysis of Oxylipins

The high-performance liquid chromatography (HPLC) and mass spectrometry methods used for the analysis of plasma oxylipins was based on methods previously described for the analysis of oxylipins in liver (24). The analysis was performed using a Shimadzu Prominence HPLC system (Shimadzu, Columbia, MD) coupled to an Applied Biosystems 4000 QTRAP (AB SCIEX, Framingham, MA). Employing dynamic multireaction monitoring (dMRM), we evaluated 60 oxylipins in a targeted approach (Supplementary Figure 1). For each compound, optimal transitions were determined by flow injection of pure standards using the optimizer application, and transitions were compared with literature when available. A detailed list of MRM transitions and experimental conditions is provided in Supplementary Table 2.

Compounds were separated using a Waters Acquity UPLC CSH C18 column (100 mm length × 2.1 mm id; 1.7 μm particle size) with an additional Waters Acquity VanGuard CSH C18 pre-column (5 mm × 2.1 mm id; 1.7 μm particle size). Column oven was set to 60°C. The mobile phase consisted of (A) water containing 0.1% acetic acid and (B) acetonitrile/isopropanol (ACN/IPA) (90/10, *v*/*v*) containing 0.1% acetic acid. Gradient elution (22) was carried out for 22 min at a flow rate of 0.15 ml min^−1^. Gradient conditions were as follows: 0–1.0 min, 0.1–25% B; 1.0–2.5 min, 25–40% B; 2.5–4.5 min, 40–42% B; 4.5–10.5 min, 42–50% B; 10.5–12.5 min, 50–65% B; 12.5–14 min, 65–75% B; 14–14.5 min, 75–85% B; 14.5–20 min, 85–95% B; 20–20.5 min, 95–95% B; 20.5–22 min, 95–25% B. A 5-μl aliquot of each sample was injected. Limits of detection (LOD) and quantification (LOQ) (Supplementary Table 1) were calculated based on one concentration point (0.1 ng μl^−1^) for each oxylipin and deuterated surrogate.

### Data Processing and Statistical Analysis

Raw data from targeted oxylipin analyses were imported into MultiQuant 3.0.2 software (AB SCIEX) in order to perform the alignment and integration of the peaks (obtaining peak areas). This software allows for the correction of metabolite intensity with the intensity of the internal standards. Data obtained with MultiQuant were imported into MarkerView 1.3.1 software (AB SCIEX) for initial data visualization (25).

Data were analyzed using SAS version 9.2 (SAS Ins. Inc., Cary, NC). Demographic and clinical characteristics of groups were compared using Fisher's exact test for binary data and *t*-test for nonbinary data. Concentrations of oxylipins were compared using Wilcoxon rank sum test. To evaluate diagnostic and predictive efficacy of oxylipins, we used logistic regression analysis and calculated receiver operating characteristic (ROC) values, including area under the curves (AUC). Our goal was to identify oxylipin panels that could achieve an ROC of 0.90 or higher. To compare diagnostic and predictive efficacy of oxylipins with current standard risk assessment tool, we compared ROC values of our best oxylipin models with those of the 10-year Framingham general CVD risk scores using the ROCCONTRAST statement in PROC LOGISTIC. We were not able to use the 10-year atherosclerotic CVD risk score of the American College of Cardiology (ACC) because 41 of 74 CAD patient scores could not be calculated. All statistical tests were two sided. Significance was declared at *P* ≤ 0.05.

## Results

### Analysis of Oxylipins

In order to achieve a representative coverage of LA-, ARA-, EPA-, and DHA-derived oxylipins and the enzymatic and nonenzymatic pathways involved in their production, a library with 39 oxylipin standards was analyzed (Supplementary Table 1). Our LC-MRM method detected all 39 oxylipins in one 22-min run (Supplementary Figure 1). Of the 39 oxylipins, 24 were consistently above the LOD and 22 oxylipins were consistently above the LOQ. Oxylipin concentrations below the LOQ were set at 80% of the lowest quantifiable sample. The library included (i) four LA-derived oxylipins (two each from CYP450 and LOX pathways), of which three [CYP450: 12,13-DiHOME, LOX: 9(S) HODE, 13(S) HODE] were above the LOQ; (ii) 14 ARA-derived oxylipins (five from COX, five from CYP450, and four from LOX pathways), of which nine (COX: thromboxane B2; CYP450: 11,12-EET, 14,15-EET, 20-HETE, 14,15-DiHET; LOX: 5-HETE, 12-HETE, 15-HETE, leukotriene B4) were above the LOQ; (iii) 10 EPA-derived oxylipins (one from COX, eight from CYP450, and one from ROS pathways), of which three (CYP450: 11,12-DiHETE, 17,18-EpETE; ROS: 8-iso PGF3a) were above the LOQ; (iv) 11 DHA-derived oxylipins (10 CYP450 and one from LOX pathways) of which seven (CYP450: 10,11-EpDPA, 19,20 EpDPA, 7,8-DiHDPA, 10,11-DiHDPA, 13,14-DiHDPA, 16,17-DiHDPA, 19,20-DiHDPA) were above the LOQ.

### Demographic, Clinical Characteristics, and Levels of Oxylipins of Adults With Diseased Coronary Arteries

Selected demographic and clinical characteristics of adults with diseased coronary arteries stratified by number of diseased arteries and adults of the same age range with a low CAD risk are listed in Table 1. Sixty-nine of 74 adults with CAD had multiple CVD risk factors (three CAD1 patients and one CAD2 patient had one CVD risk factor and one CAD2 patient had no CVD risk factor). Almost all adults with CAD had hypertension and hypercholesterolemia. Most adults with CAD were on aspirin, were overweight or obese, or had a history of smoking. About half adults with CAD had diabetes or a family history of CVD. Demographic and clinical characteristics of adults with CAD had a limited efficacy to diagnose number of diseased arteries. The 10-year Framingham general CVD risk score and the number of CAD risk factors increased with the number of diseased arteries; specifically, adults with multiple diseased arteries were more likely to be male, were overweight or obese, former smokers, or had lower plasma HDL cholesterol concentrations.

**Table 1:** Demographic and clinical characteristics of adults with diseased coronary arteries (≥70% stenosis) and adults of the same age range with a low coronary artery disease (CAD) risk.

**Characteristics**	**Low**	**Number of diseased arteries**			**Contrast**
	**CAD risk** ** (*n* = 23)**	**1** ** (*n* = 31)**	**2** ** (*n* = 23)**	**3** ** (*n* = 20)**	**CAD1/2 vs. CAD3**
	**Mean ± STD**	**Mean ± STD**	**Mean ± STD**	**Mean ± STD**	***P-*value**
Age (year)	49 ± 10^b^	65 ± 9^a^	67 ± 12^a^	66 ± 1^a^	0.89
Male [*n* (%)]	4 (17)^c^	17 (55)^b^	19 (83)^a^	17 (85)^a^	0.15
BMI (kg/m^2^)	28.3 ± 6.7^ab^	28.1 ± 4.9^b^	31.0 ± 9.3^ab^	31.7 ± 6.1^a^	0.20
Overweight	3 (13)^ab^	15 (48)^a^	9 (39)^ab^	8 (40)^ab^	0.80
Obese	9 (39)	8 (26)	10 (43)	10 (50)	0.28
**Blood pressure (mmHg)**					
Systolic	124 ± 9	131 ± 20	133 ± 17	129 ± 17	0.58
Diastolic	79 ± 6^a^	71 ± 13^b^	69 ± 11^b^	70 ± 11^b^	0.89
**Plasma**					
Triacylglycerol (mg/dl)	88 ± 38^b^	136 ± 72^a^	165 ± 106^a^	218 ± 326^ab^	0.40
Total cholesterol (mg/dl)	195 ± 32	182 ± 49^a^	156 ± 33^b^	177 ± 60^ab^	0.62
HDL cholesterol (mg/dl)	65 ± 13^a^	52 ± 16^b^	46 ± 14^b^	40 ± 12^c^	0.03
LDL cholesterol (mg/dl)	126 ± 28^a^	101 ± 35^b^	76 ± 25^b^	104 ± 48^ab^	0.28
Hba1c (mmol/mol)	5.3 ± 0.4^b^	6.1 ± 1.1^a^	6.5 ± 1.3^a^	6.3 ± 1.1^a^	0.90
Medication	0^b^	30 (97)^a^	21 (91)^a^	18 (90)^a^	0.61
Blood pressure [total, *n* (%)]	0^b^	24 (77)^b^	21 (91)^ab^	20 (100)^a^	0.10
ACE inhibitor [*n* (%)]	0^b^	10 (32)^a^	8 (35)^a^	5 (25)^a^	0.58
Angiotension receptor blocker	0^b^	5 (16)^ab^	3 (13)^ab^	5 (25)^a^	0.32
Beta blocker [*n* (%)]	0^b^	18 (58)^a^	19 (83)^a^	13 (65)^a^	0.79
Calcium channel blocker [*n* (%)]	0^b^	7 (23)^a^	4 (17)^ab^	5 (25)^a^	0.75
Diabetes [total, *n* (%)]	0^b^	6 (19)^a^	6 (26)^a^	6 (30)^a^	0.55
Oral hyperglycemia [*n* (%)]	0^b^	3 (10)^ab^	3 (13)^ab^	4 (20)^a^	0.44
Insulin [*n* (%)]	0	3 (10)	3 (13)	2 (10)	1
**Hyperlipidemia (total)**					
Statin [*n* (%)]	0^b^	24 (77)^a^	16 (70)^a^	13 (65)^a^	0.56
Aspirin [*n* (%)]	0^b^	23 (74)^a^	19 (83)^a^	15 (75)^a^	0.77
**CVD risk factors**					
Tobacco use [*n* (%)]					
Former	6 (26)	8 (26)	9 (39)	6 (30)	1
Active	0^b^	4 (13)^ab^	2 (9)^ab^	5 (25)^a^	0.15
**History of [*****n*** **(%)]**					
Hypertension	2 (9)^b^	25 (81)^a^	22 (96)^a^	20 (100)^a^	0.18
Diabetes	0^b^	8 (26)^a^	8 (49)^a^	9 (45)^a^	0.27
Hypercholesterolemia	0^b^	30 (97)^a^	21 (91)^a^	19 (95)^a^	1
CVD in family	0^b^	14 (45)^a^	12 (52)^a^	11 (55)^a^	0.79
Total risk (1–5)	0^c^	2.6 ± 0.9^b^	2.8 ± 0.9^ab^	3.2 ± 0.8^a^	0.04
Framingham 10-year CVD risk (%)	4.0 ± 2.3^c^	21.6 ± 16.2^b^	29.4 ± 17.5^ab^	35.7 ± 19.7^a^	0.04
ACC 10-year ASCVD risk (%)	1.8 ± 1.2^b^	15.6 ± 11.7^a^	18.6 ± 7.5^a^	25.7 ± 12.3^a^	0.05

*The superscripts denote whether averages of specific groups in a row differed at P ≤ 0.05. If none of the groups differed from each other, no superscripts are shown. If two groups differed from each other, the significantly higher group got a superscript a, and the significantly lower group got a superscript b, and the group that did not significantly differ from the higher or the lower group got a superscript ab. If three groups differed from each other, the significantly highest group got a superscript a, the significantly lower group got a superscript b, and the group that was significantly lower than the group with a superscript b, got a, c. Quantitative data were compared using Student's t-test. Proportions were compared using Fisher's exact test*.

*ACE, angiotension converting enzyme; ACC, American College of Cardiology; ASCVD, atherosclerotic cardiovascular disease*.

Ten of 22 (45%) individual oxylipin concentrations decreased with greater number of diseased arteries by at least 10%; six individual oxylipins (27%) had significantly lower concentrations in adults with three vs. one diseased artery (Table 2). For pattern detection, oxylipins were grouped in Table 3 by (1) FA precursors (i.e., LA, ARA, EPA, and DHA), oxylipin groups (i.e., mid-chain HODE, EET, mid-chain HETE, EpDPA, DiHDPA), (2) enzymes involved in their synthesis [i.e., oxygenation of PUFAs by LOX followed by reduction or alternatively hydroxylation of PUFAs by CYP1B1; oxidation of PUFAs by CYP450 followed by hydroxylation of oxidized PUFAs by soluble epoxide hydrolase (sEH)], and (3) based on enzymatic product to substrate ratio (i.e., hydroxylation of 10,11-EpDPA to 10,11-DiHDPA, 14,15-EET to 14,15-DiHET, or 19,20-EpDPA to 19,20-DiHDPA by sEH).

**Table 2:** Plasma oxylipin concentrations of adults with diseased coronary arteries (≥70% stenosis) and adults of the same age range with a low coronary artery disease (CAD) risk.

**Oxylipins (nM)**	**Low**	**Number of diseased arteries**			**Contrast**
	**CAD risk** ** (*n* = 23)**	**1** ** (*n* = 31)**	**2** ** (*n* = 23)**	**3** ** (*n* = 20)**	**CAD1/2 vs. CAD3**
	**Median** ** (IQR)**	**Median** ** (IQR)**	**Median** ** (IQR)**	**Median** ** (IQR)**	***P*-value**
12,13-DiHOME	8.71^a^ (5.27, 11.8)	6.97^ab^ (4.76, 8.85)	5.59^bc^ (4.07, 8.45)	5.11^c^ (4.17, 5.89)	0.07
13(S)-HODE	29.1^b^ (26.7, 33.8)	37.6^a^ (25.5, 49.9)	34.1^ab^ (23.4, 41.7)	31.0^ab^ (24.4, 43.2)	0.64
9(S)-HODE	19.0 (17.6, 21.6)	22.3 (16.8, 28.8)	20.3 (16.9, 25.0)	20.5 (14.9, 26.7)	0.64
Leukotriene B4	0.19^ab^ (0.15, 0.24)	0.23^a^ (0.18, 0.28)	0.24^a^ (0.16, 0.27)	0.18^b^ (0.15, 0.21)	0.01
Thromboxane B2	0.05 (0.03, 0.08)	0.03 (0.02, 0.05)	0.04 (0.02, 0.04)	0.04 (0.03, 0.06)	0.10
20-HETE	9.18 (7.04, 11.2)	8.60 (7.39, 10.4)	9.37 (7.35, 10.7)	8.43 (6.98, 9.52)	0.36
14,15-EET	0.27^b^ (0.18, 0.34)	0.34^a^ (0.28, 0.45)	0.22^b^ (0.18, 0.36)	0.35^a^ (0.23, 0.40)	0.70
11,12-EET	0.25^b^ (0.19, 0.30)	0.32^a^ (0.26, 0.41)	0.32^a^ (0.27, 0.37)	0.35^a^ (0.28, 0.46)	0.35
14,15-DiHET	0.98^ab^ (0.78, 1.13)	1.12^a^ (0.93, 1.23)	0.99^ab^ (0.84, 1.17)	0.90^b^ (0.78,1.09)	0.08
5-HETE	2.69^b^ (2.30, 3.14)	4.63^a^ (3.37, 7.29)	4.37^a^ (2.87, 6.86)	3.87^ab^ (2.21, 6.65)	0.38
12-HETE	2.41^b^ (1.13, 4.19)	8.14^a^ (5.60, 20.8)	11.2^a^ (6.51, 19.6)	7.92^a^ (2.78, 12.0)	0.10
15-HETE	1.18^b^ (1.02,1.53)	2.41^a^ (1.84, 3.29)	2.39^a^ (1.73, 3.49)	2.26^a^ (1.47, 3.11)	0.47
8-iso PGF3a	0.93^a^ (0.49, 1.58)	0.99^a^ (0.55, 1.95)	0.87^ab^ (0.40, 2.70)	0.58^b^ (0.37, 0.81)	0.02
17,18-EpETE	0.29 (0.23, 0.35)	0.26 (0.20, 0.35)	0.26 (0.21, 0.34)	0.29 (0.24, 0.33)	0.51
11,12-DiHETE	0.08 (0.04, 0.11)	0.06 (0.04, 0.12)	0.09 (0.06, 0.12)	0.05 (0.01, 0.09)	0.07
19,20-EpDPA	0.53 (0.32, 0.95)	0.59 (0.43, 0.91)	0.50 (0.40, 0.76)	0.60 (0.48, 0.83)	0.37
10,11-EpDPA	0.11 (0.05, 0.16)	0.11 (0.07, 0.14)	0.12 (0.09, 0.20)	0.11 (0.07, 0.15)	0.16
19,20-DiHDPA	2.22^a^ (1.40, 2.83)	1.92^a^ (1.19, 2.76)	1.69^ab^ (1.00, 2.63)	0.60^b^ (0.48, 0.83)	0.04
13,14-DiHDPA	0.14^a^ (0.07, 0.20)	0.11^ab^ (0.07, 0.14)	0.09^ab^ (0.07, 0.19)	0.09^b^ (0.06, 0.12)	0.39
16,17-DiHDPA	0.24^ab^ (0.15, 0.34)	0.24^a^ (0.16, 0.28)	0.19^ab^ (0.14, 0.27)	0.17^b^ (0.14, 0.21)	0.04
10,11-DiHDPA	0.09 (0.06, 0.15)	0.11 (0.08, 0.17)	0.09 (0.06, 0.18)	0.08 (0.05, 0.11)	0.06
7,8-DiHDPA	0.12 (0.08, 0.14)	0.13 (0.09, 0.16)	0.11 (0.09, 0.17)	0.12 (0.10, 0.18)	0.74

*The superscripts denote whether averages of specific groups in a row differed at P ≤ 0.05 using Kruskal–Wallis test. If none of the groups differed from each other, no superscripts are shown. If two groups differed from each other, the significantly higher group got a superscript a, and the significantly lower group got a superscript b, and the group that did not significantly differ from the higher or the lower group got a superscript ab. If three groups differed from each other, the significantly highest group got a superscript a, the significantly lower group got a superscript b, and the group that was significantly lower than the group with a superscript b, got a, c*.

*DiHOME, dihydroxy-octadecenoic acid; HODE, hydroxy-octadecadienoic acid; HETE, hydroxy-eicosatetraenoic acid; EET, epoxy-eicosatrienoic acid; DiHET, dihydroxy-eicosatrienoic acid; EpDPA, epoxy-docosapentaenoic acid; DiHDPA, dihydroxy-docosapentaenoic acid*.

**Table 3:** Plasma concentrations of oxylipin groups in adults with diseased coronary arteries (≥70% stenosis) and adults of the same age range with a low coronary artery disease (CAD) risk.

**Oxylipins (nM)**	**Low**	**Number of diseased arteries**			**Contrast**
	**CAD risk** ** (*n* = 23)**	**1** ** (*n* = 31)**	**2** ** (*n* = 23)**	**3** ** (*n* = 20)**	**CAD1/2 vs. CAD3**
	**Median** ** (IQR)**	**Median** ** (IQR)**	**Median** ** (IQR)**	**Median** ** (IQR)**	***P*-value**
Total	80.3^b^ (75.9, 102)	109^a^ (85.8, 171)	97.9^ab^ (82.0, 116)	85.4^b^ (74.4, 108)	0.05
**Fatty acid precursor**					
C18:2 derived	47.7^b^ (43.9, 54.7)	59.7^a^ (41.7, 76.7)	54.1^ab^ (47.4, 63.0)	55.5^ab^ (45.0, 73.4)	0.64
C20:4 derived	18.5^b^ (14.8, 21.3)	28.0^a^ (22.0, 40.7)	28.6^a^ (22.6, 42.8)	24.4^a^ (18.5, 28.9)	0.11
C20:5 derived	1.35^a^ (0.91, 2.01)	1.41^a^ (0.83, 2.48)	1.14^ab^ (0.76, 3.08)	0.94^b^ (0.64, 1.24)	0.04
C22:6 derived	3.36^a^ (2.26, 5.06)	3.36^ab^ (2.16, 4.25)	3.14^ab^ (2.01, 4.03)	2.59^b^ (2.20, 3.19)	0.18
**Oxylipin group**					
Mid-chain HODE	47.7^b^ (44.7, 54.7)	59.7^a^ (41.7, 76.7)	54.1^ab^ (47.4, 63.0)	51.2^ab^ (40.1, 68.7)	0.64
EET	1.45^b^ (1.28, 1.68)	1.77^a^ (1.45, 2.16)	1.63^ab^ (1.32, 1.87)	1.51^ab^ (1.39, 1.89)	0.47
Mid-chain HETE	6.34^b^ (5.06, 9.50)	17.1^a^ (12.1, 27.0)	19.0^a^ (10.6, 32.4)	13.2^a^ (8.07, 19.8)	0.11
EpDPA	0.67 (0.36, 1.06)	0.75 (0.51, 1.13)	0.66 (0.50, 0.94)	0.74 (0.57, 0.99)	0.72
DiHDPA	2.84^a^ (1.89, 3.70)	2.50^a^ (1.63, 3.45)	2.34^ab^ (1.37, 3.28)	1.89^b^ (1.37, 2.33)	0.04
**Enzyme products**					
LOX/CYP1B1 products	55.8^b^ (53.0, 68.0)	78.9^a^ (60.7, 114)	79.1^a^ (59.3, 92.3)	68.8^ab^ (55.4, 89.4)	0.27
LOX12-15 products	32.9^b^ (19.7, 40.4)	47.9^a^ (37.2, 72.2)	53.8^a^ (31.5, 62.5)	43.5^a^ (31.6, 55.1)	0.26
LOX5 products	21.9^b^ (20.1, 25.6)	27.2^a^ (21.0, 37.3)	26.5^ab^ (19.9, 30.2)	25.1^ab^ (18.4, 31.4)	0.55
CYP epoxides	1.44 (1.19, 1.90)	1.70 (1.41, 2.19)	1.56 (1.22, 2.14)	1.94 (1.32, 2.11)	0.86
Hydroxylated CYP epoxides	11.8^a^ (8.61, 20.8)	10.0^ab^ (8.02, 14.1)	9.50^bc^_(_7.35, 11.9)	7.77^c^ (6.40, 9.21)	0.008
**sEH product to substrate ratios**					
10,11-DiHDPA/10,11-EpDPA	0.90^a^ (0.78, 1.30)	0.81^b^ (0.63, 0.93)	0.79^b^ (0.59, 1.04)	0.71^b^ (0.52, 0.92)	0.21
14,15-DiHET/14,15-EET	4.03^a^ (2.91, 5.34)	2.88^b^ (2.39, 4.13)	3.76^a^ (2.63, 5.37)	2.56^b^ (2.29, 4.17)	0.08
19,20-DiHDPA/19,20-EpDPA	4.26^a^ (2.48, 6.55)	2.97^a^ (2.19, 3.92)	2.95^a^ (2.13, 4.77)	1.91^b^ (1.58, 2.98)	0.002

*The superscripts denote whether averages of specific groups in a row differed at P ≤ 0.05 using Kruskal–Wallis test. If none of the groups differed from each other, no superscripts are shown. If two groups differed from each other, the significantly higher group got a superscript a, and the significantly lower group got a superscript b, and the group that did not significantly differ from the higher or the lower group got a superscript ab. If three groups differed from each other, the significantly highest group got a superscript a, the significantly lower group got a superscript b, and the group that was significantly lower than the group with a superscript b, got a, c*.

*HODE, hydroxy-octadecadienoic acid; HETE, hydroxy-eicosatetraenoic acid; EET, epoxy-eicosatrienoic acid; DiHET, dihydroxy-eicosatrienoic acid; EpDPA, epoxy-docosapentaenoic acid; DiHDPA, dihydroxy-docosapentaenoic acid; LOX, lipoxygenase; CYP, cytochrome P450; sEH, soluble epoxide hydrolases*.

Total oxylipin concentrations significantly declined with number of diseased arteries, specifically omega-3 FA-derived oxylipins and within those hydroxylated DHA-epoxide DiHDPAs, which are generated by hydroxylation of oxidized PUFAs by sEH (Table 3). The strongest decline was observed for hydroxylation of 19,20-EpDPA to 19,20-DiHDPA.

Low CAD risk adults had lower total oxylipin concentrations than adults with CAD, specifically omega-6 FA-derived oxylipins and within those mid-chain HETEs (Tables 2, 3). These include the following three oxylipins that were significantly lower than in each CAD group: 11,12-EET, 12-HETE, and 15-HETE. We also observed less hydroxylation of 10,11-EpDPA to 10,11-DiHDPA, which is generated by sEH. Concentrations of LA-derived 12,13-DiHOME and DHA-derived DiHDPAs, specifically 19,20-DiHDPA and 16,17-DiHDPA, decreased gradually from adults with low CAD risk to those with three diseased arteries.

### Diagnostic Efficacy of Oxylipins

**Figure 3:**
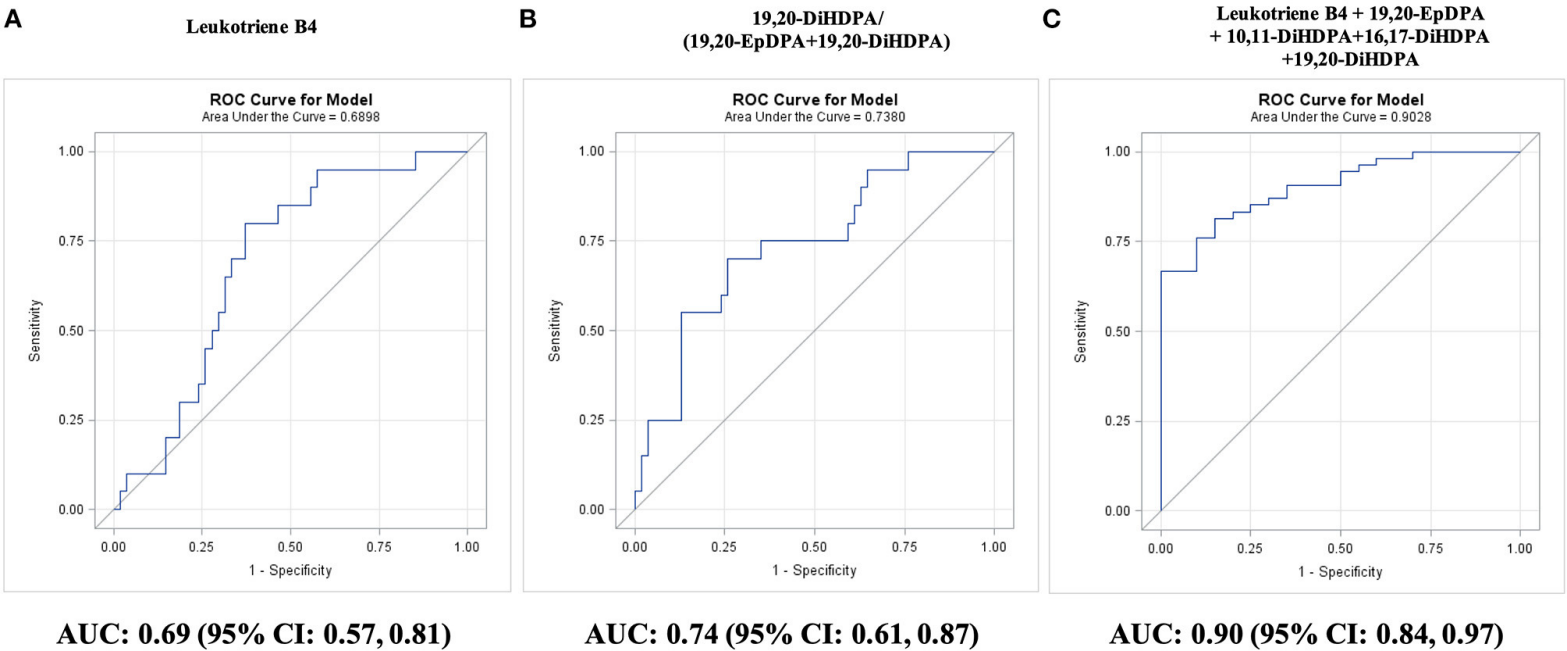
Diagnosis of a number of diseased coronary arteries in adults with diseased coronary arteries (≥70% stenosis; *n* = 74), as shown by receiver operating characteristic (ROC) curves: **(A)** best single oxylipin model; **(B)** best single oxylipin group model; and **(C)** smallest oxylipin panel model achieving AUC ≥ 0.90.

Differences in plasma oxylipin concentrations were noted primarily between two and three diseased vessels. Among individual oxylipins, ARA-derived leukotriene B4 discriminated best three vs. less diseased arteries (AUC: 0.69; 95% CI: 0.57–0.81; *P* = 0.003; Figure 3A). Leukotriene B4 concentrations ≤0.21 nM diagnosed three diseased arteries in 80% of CAD3 adults and less diseased arteries in 65% CAD1 adults, 61% CAD2 adults, and 43% adults with low CAD risk.

Significant AUC values were also observed for EPA-derived 8-iso PGF3a (AUC: 0.67; 95% CI: 0.54–0.80; *P* = 0.009), three DHA-derived DiHDPA 19,20-DiHDPA (AUC: 0.66; 95% CI: 0.54–0.78; *P* = 0.01), 16,17-DiHDPA (AUC: 0.65; 95% CI: 0.52–0.79; *P* = 0.02), 10,11-DiHDPA (AUC: 0.64; 95% CI: 0.51–0.78; *P* = 0.04), and LA-derived 12,13-DiHOME (AUC: 0.64; 95% CI: 0.51–0.77; *P* = 0.04).

Among oxylipin groups and ratios, three diseased arteries were best diagnosed by the 19,20-DiHDPA fraction of the sum of 19,20-EpDPA and 19,20-DiHDPA (AUC: 0.74; 95% CI: 0.61–0.87; *P* = 0.0003; Figure 3B). A fraction of <72% diagnosed three diseased arteries in 70% of CAD3 adults and less diseased arteries in 74% CAD1 adults, 70% CAD2 adults, and 78% adults with low CAD risk. Adding 8-iso PGF3a to the fraction improved diagnosis of three diseased arteries to 80% but decreased diagnosis of less diseased arteries to 60% in CAD2 adults. An oxylipin panel of leukotriene B4, 19,20-EpDPA, 19,20-DiHDPA, 13,14-DiHDPA, and 10,11-DiHDPA diagnosed three diseased arteries in all CAD3 adults and less diseased arteries in 70% CAD1 and CAD2 adults (AUC: 0.90; 95% CI: 0.84–0.97; *P* < 0.0001; Figure 3C). The oxylipin panel improved (*P* = 0.02) diagnosis of three diseased arteries compared with the 10-year Framingham general CVD risk score (AUC: 0.68; 95% CI: 0.52–0.83; *P* = 0.02).

### Prediction of Outcomes in Adults With Diseased Coronary Arteries

Adults with CAD were followed up until November 2019 for a median of 5 years (range: 25–84 months) and adverse events were recorded (i.e., coronary stent placement; CABG surgery; death). Ten participants (three women and seven men; median age: 61 years; range: 51–81 years) were lost to follow-up (Figure 2). Given the degree of stenosis, 52 of 64 adults with CAD underwent CABG surgery within 3 months of the angiography (CAD1: 19 of 28; CAD2: 16 of 19; CAD3: 17 of 17): 28 had a CABG surgery (CAD1: 5 of 19; CAD2: 10 of 16; CAD3: 13 of 17) and 26 had a coronary stent placement (CAD1: 16; CAD2: 6; CAD3: 4). Adults with multiple diseased coronary arteries were more likely to receive a CABG. Of the remaining 12 adults with CAD, seven had no further event, two adults with one diseased artery received a coronary artery stent during follow-up, and three died (CAD1: 2; CAD2: 1). In addition, four CAD adults that had undergone open-heart surgery within 3 months (two stents and two CABG) died during follow-up (CAD1: 1; CAD2: 0; CAD3: 3). Survival was not linked to the number of diseased coronary arteries.

Table 4 lists selected demographic and clinical characteristics of adults with diseased coronary arteries (≥70% stenosis) based on outcomes during follow-up. Survival was linked to lower systolic blood pressure or being a male, whereas survival without CABG was linked to higher plasma triacylglycerol concentrations. Unfavorable outcomes were linked to elevated oxylipin concentrations (Table 5), specifically omega-6 FA-derived oxylipins and within those LA-derived mid-chain HODEs and ARA-derived mid-chain HETEs, which are either generated by oxygenation of lipoxygenases or hydroxylation of CYP1B1 (Table 6). Concentrations of LA-derived 9(S)-HODE and 13(S)-HODE and ARA-derived thromboxane B2, 5-HETE, and 15-HETE increased gradually from stent placement to CABG to death. In contrast, concentrations of EPA-derived 8-iso PGF3α were lower with unfavorable outcomes.

**Table 4:** Demographic and clinical characteristics of adults with diseased coronary arteries (≥70% stenosis) stratified by outcome during 5-year follow-up.

**Characteristics**	**Outcome during follow-up**				**Contrasts**		
	**No event** ** (*n* = 7)**	**Stent** ***(n* = 24)**	**CABG** ***(n* = 26)**	**Death** ***(n* = 7)**	**No event** ** vs. others**	**CABG/death** ** vs. others**	**Death** ** vs. Others**
	**Mean ± STD**	**Mean ± STD**	**Mean ± STD**	**Mean ± STD**	***P*-value**	***P*-value**	***P*-value**
Age (year)	65 ± 8	68 ± 8	67 ± 10	60 ± 17	0.71	0.52	0.31
Male [*n* (%)]	3 (43)^bc^	19 (79)^ab^	22 (85)^a^	2 (29)^c^	0.08	1	0.02
BMI (kg/m^2^)	30.2 ± 8.5	29.9 ± 8.8	28.3 ± 3.3	29.9 ± 5.6	0.70	0.46	0.78
Overweight [*n* (%)]	1 (14)^b^	9 (38)^ab^	16 (62)^a^	4 (57)^ab^	0.11	0.03	0.70
Obese [*n* (%)]	3 (43)	9 (38)	7 (27)	2 (29)	0.67	0.43	1
**Blood pressure (mmHg)**							
Systolic	130 ± 16^ab^	130 ± 16^b^	130 ± 19^ab^	147 ± 15^a^	0.81	0.42	0.02
Diastolic	65 ± 12	72 ± 10	70 ± 11	67 ± 8	0.20	0.63	0.42
**Plasma**							
Triacylglycerol (mg/dl)	197 ± 131	167 ± 100	123 ± 59	89 ± 42	0.29	0.02	0.13
Total cholesterol (mg/dl)	174 ± 44	187 ± 50	162 ± 42	153 ± 46	0.91	0.06	0.29
HDL cholesterol (mg/dl)	48 ± 14	49 ± 12	44 ± 18	53 ± 14	0.94	0.51	0.39
LDL cholesterol (mg/dl)	92 ± 35	104 ± 37	94 ± 41	73 ± 39	0.79	0.29	0.17
Hba1c (mmol/mol)	5.9 ± 0.8	6.9 ± 0.4	6.2 ± 1.3	6.5 ± 2.5	0.61	0.74	0.95
Medication [*n* (%)]	7 (100)	23 (96)	25 (96)	7 (100)	1	1	1
Blood pressure (total)	6 (86)	22 (92)	22 (85)	6 (86)	1	0.71	1
ACE inhibitor	2 (29)	10 (42)	6 (23)	2 (29)	1	0.28	1
AR blocker	2 (29)	4 (17)	3 (12)	2 (29)	0.59	0.75	0.59
Beta blocker	3 (43)^b^	20 (83)^a^	17 (65)^ab^	4 (57)^ab^	0.19	0.43	0.67
Calcium channel blocker	3 (43)	4 (17)	6 (23)	1 (14)	0.17	1	1
Diabetes (total)	2 (29)	5 (21)	6 (23)	3 (43)	1	0.78	0.35
Oral hyperglycemia	2 (29)	3 (13)	3 (12)	1 (14)	0.25	0.73	1
Insulin	0	2 (8)	3 (12)	2 (29)	1	0.43	0.17
**Hyperlipidemia (total)**							
Statin	6 (86)	19 (79)	20 (77)	5 (71)	1	0.77	0.64
Aspirin	6 (86)	20 (83)	21 (81)	5 (71)	1	0.75	0.61
**CAD risk factors**							
**Tobacco use [*****n*** **(%)]**							
Former	0	9 (40)	8 (31)	1 (14)	0.18	1	0.12
Active	1 (14)	2 (8)	4 (15)	3 (43)	1	0.30	0.07
**History of [*****n*** **(%)]**							
Hypertension	6 (86)	22 (92)	22 (85)	7 (100)	0.57	1	1
Diabetes	3 (43)	6 (25)	8 (31)	4 (57)	0.67	0.60	0.20
Hypercholesterolemia	7 (100)	24 (100)	23 (88)	7 (100)	1	0.49	1
CVD in family	4 (57)	12 (50)	13 (50)	3 (43)	1	1	1
Total risk (1–5)	3.0 ± 0.8^ab^	2.8 ±0.7^b^	2.7 ± 1.0^ab^	3.4 ± 1.0^a^	0.60	0.85	0.06
Framingham 10-year CVD risk (%)	23.0 ± 18.8	28.0 ± 17.9	30.1 ± 18.3	28.4 ± 22.2	0.41	0.53	0.98
ACC 10-year ASCVD risk (%)	17.5 ± 11.9	18.9 ± 10.1	20.9 ± 13.8	9.0	0.77	0.77	ND

*The superscripts denote whether averages of specific groups in a row differed at P ≤ 0.05. If none of the groups differed from each other, no superscripts are shown. If two groups differed from each other, the significantly higher group got a superscript a, and the significantly lower group got a superscript b, and the group that did not significantly differ from the higher or the lower group got a superscript ab. If three groups differed from each other, the significantly highest group got a superscript a, the significantly lower group got a superscript b, and the group that was significantly lower than the group with a superscript b, got a, c. Data were compared using Student's t-test. Proportions were compared using Fisher's exact test*.

*CABG, coronary artery bypass grafting; ACE, angiotension converting enzyme; AR, angiotension receptor; ACC, American College of Cardiology; ASCVD, Atherosclerotic Cardiovascular Disease*.

**Table 5:** Plasma oxylipin concentrations of adults with diseased coronary arteries (≥70% stenosis) stratified by outcome during 5-year follow-up.

**Oxylipins (nM)**	**Outcome during follow-up**				**Contrasts**		
	**No event** ** (*n* = 7)**	**Stent** ***(n* = 24)**	**CABG** ***(n* = 26)**	**Death** ***(n* = 7)**	**No event** ** vs. others**	**CABG/death** ** vs. others**	**Death** ** vs. others**
	**Median** ** (IQR)**	**Median** ** (IQR)**	**Median** ** (IQR)**	**Median** ** (IQR)**	***P-*value**	***P-*value**	***P-*value**
12,13-DiHOME	5.05 (3.81, 6.68)	5.71 (4.25, 7.64)	5.47 (4.29, 8.56)	5.69 (5.09, 6.93)	0.38	0.66	0.82
13(S)-HODE	33.9^cb^ (18.5, 37.8)	27.4^b^ (17.6, 40.2)	34.1^b^ (25.2, 42.2)	46.2^a^ (42.6, 58.9)	0.71	0.04	0.007
9(S)-HODE	19.4^cb^ (12.2, 25.8)	20.0^b^ (11.5, 22.4)	20.9^b^ (17.1, 25.8)	30.6^a^ (21.9, 56.9)	0.65	0.03	0.01
Leukotriene B4	0.22 (0.15, 0.24)	0.24 (0.17, 0.28)	0.22 (0.17, 0.25)	0.19 (0.16, 0.22)	0.47	0.36	0.14
Thromboxane B2	0.04^ab^ (0.02, 0.11)	0.03^b^ (0.02, 0.04)	0.04^a^ (0.02, 0.06)	0.04^a^ (0.04, 0.12)	0.89	0.04	0.08
20-HETE	8.03 (7.75, 9.93)	8.57 (6.88, 10.2)	8.62 (8.11, 10.4)	8.63 (5.66, 9.20)	0.54	0.44	0.58
14,15-EET	0.31 (0.22, 0.41)	0.28 (0.20, 0.38)	0.36 (0.22, 0.46)	0.29 (0.18, 0.38)	0.89	0.20	0.67
11,12-EET	0.35^ab^ (0.27, 0.41)	0.29^b^ (0.23, 0.38)	0.36^a^ (0.22, 0.46)	0.32^ab^ (0.24, 0.39)	0.72	0.08	0.92
14,15-DiHET	1.04 (0.87, 1.23)	0.97 (0.74, 1.13)	1.05 (0.89, 1.19)	1.03 (0.86, 1.18)	0.79	0.38	0.97
5-HETE	5.87^a^ (4.12, 11.08)	3.68^b^ (2.28, 5.00)	4.50^ab^ (2.89, 5.98)	5.00^a^ (4.43, 8.63)	0.07	0.31	0.04
12-HETE	8.14 (5.42, 11.2)	7.00 (4.60, 10.0)	10.9 (5.54, 20.8)	11.1 (5.48, 25.5)	0.97	0.09	0.38
15-HETE	2.92^ab^ (1.84, 2.97)	1.86^b^ (1.48, 2.34)	2.52^a^ (2.05, 3.19)	2.44^a^ (2.13, 4.17)	0.51	0.03	0.27
8-iso PGF3a	0.60^b^ (0.29, 0.99)	1.48^a^ (0.60, 14.6)	0.64^ab^ (0.43, 1.95)	0.39^b^ (0.34, 0.72)	0.28	0.08	0.06
17,18-EpETE	0.22 (0.19, 0.33)	0.28 (0.22, 0.35)	0.29 (0.19, 0.35)	0.31 (0.24, 0.39)	0.42	0.68	0.34
11,12-DiHETE	0.06 (0.04, 0.19)	0.05 (0.03, 0.13)	0.08 (0.04,0.12)	0.06 (0.04, 0.09)	0.67	0.81	0.57
19,20-EpDPA	0.70 (0.47, 1.04)	0.51 (0.28, 0.76)	0.64 (0.46, 0.83)	0.49 (0.40-0.56)	0.33	0.54	0.38
10,11-EpDPA	0.12 (0.10, 0.38)	0.10 (0.06, 0.19)	0.13 (0.06, 0.20)	0.09 (0.08, 0.11)	0.35	0.93	0.60
19,20-DiHDPA	1.61 (1.26, 2.81)	1.63 (0.91, 2.36)	1.44 (0.97, 2.18)	1.78 (1.19, 2.73)	0.63	0.64	0.45
13,14-DiHDPA	0.07 (0.06, 0.21)	0.10 (0.06, 0.15)	0.11 (0.08, 0.15)	0.08 (0.04, 0.12)	0.97	0.91	0.20
16,17-DiHDPA	0.20 (0.15, 0.41)	0.20 (0.13, 0.28)	0.18 (0.14, 0.24)	0.20 (0.15, 0.24)	0.43	0.43	0.99
10,11-DiHDPA	0.11 (0.04, 0.33)	0.09 (0.05, 0.13)	0.09 (0.06, 0.16)	0.08 (0.06, 0.11)	0.58	0.79	0.48
7,8-DiHDPA	0.15 (0.08, 0.20)	0.12 (0.08, 0.15)	0.12 (0.09, 0.17)	0.14 (0.11, 0.18)	0.48	0.82	0.67

*The superscripts denote whether averages of specific groups in a row differed at P ≤ 0.05 using Kruskal–Wallis test. If none of the groups differed from each other, no superscripts are shown. If two groups differed from each other, the significantly higher group got a superscript a, and the significantly lower group got a superscript b, and the group that did not significantly differ from the higher or the lower group got a superscript ab. If three groups differed from each other, the significantly highest group got a superscript a, the significantly lower group got a superscript b, and the group that was significantly lower than the group with a superscript b, got a, c*.

*CABG, coronary artery bypass grafting; DiHOME, dihydroxy-octadecenoic acid; HODE, hydroxy-octadecadienoic acid; HETE, hydroxy-eicosatetraenoic acid; EET, epoxy-eicosatrienoic acid; DiHET, dihydroxy-eicosatrienoic acid; EpDPA, epoxy-docosapentaenoic acid; DiHDPA, dihydroxy-docosapentaenoic acid*.

**Table 6:** Plasma concentrations of oxylipin groups in adults with diseased coronary arteries (≥70% stenosis) stratified by outcome during 5-year follow-up.

**Oxylipins (nM)**	**Outcomes during follow-up**				**Contrasts**		
	**No event** ***(n* = 7)**	**Stent** ***(n* = 24)**	**CABG** ** (*n* = 26)**	**Death** ***(n* = 7)**	**No event** ** vs. others**	**CABG/death** ** vs. others**	**Death** ** vs. others**
	**Media**n ** (IQR)**	**Median** ** (IQR)**	**Median** ** (IQR)**	**Median** ** (IQR)**	***P*-value**	***P*-value**	***P*-value**
Total	97.8^ab^ (62.2, 112)	86.4^b^ (69.4, 132)	103^ab^ (81.4, 116)	119^a^ (97.4, 180)	0.71	0.16	0.05
**Fatty acid precursor**							
C18:2 derived	59.7^b^ (30.7, 62.4)	47.6^b^ (28.9, 62.5)	54.9^b^ (45.9, 65.8)	76.7^a^ (64.5, 135)	0.76	0.04	0.004
C20:4 derived	28.0^ab^ (24.9, 35.6)	23.3^b^ (18.3, 30.5)	28.4^a^ (23.6, 41.1)	29.0^a^ (27.3, 44.9)	0.51	0.03	0.19
C20:5 derived	0.87^ab^ (0.67, 1.37)	1.95^a^ (0.87, 14.9)	0.97^ab^ (0.74, 2.48)	0.86^b^ (0.66, 1.07)	0.31	0.13	0.17
C22:6 derived	3.02 (2.25, 5.86)	2.95 (1.48, 4.21)	2.58 (2.13, 3.93)	2.96 (2.13, 3.66)	0.58	0.80	0.84
**Oxylipin group**							
Mid-chain HODE	59.7^b^ (30.7, 62.4)	47.6^b^ (28.9, 62.5)	54.9^b^ (45.9, 65.8)	76.7^a^ (64.5, 135)	0.76	0.04	0.004
EET	1.71 (1.45, 1.80)	1.51 (1.37, 1.83)	1.75 (1.44, 2.25)	1.46 (1.32, 1.99)	0.84	0.20	0.76
Mid-chain HETE	17.4^a^ (15.2, 21.4)	12.9^b^ (10.1, 18.4)	18.0^ab^ (11.5, 30.8)	17.9^a^ (17.1, 34.1)	0.36	0.08	0.17
EpDPA	0.82 (0.53, 1.42)	0.63 (0.33, 0.92)	0.77 (0.57, 0.97)	0.56 (0.50, 0.75)	0.32	0.59	0.35
DiHDPA	2.07 (1.72, 4.05)	2.13 (1.24, 3.01)	1.89 (1.35, 2.83)	2.26 (1.58, 3.27)	0.64	0.67	0.61
**Enzyme products**							
LOX/CYP1B1 products	77.7^ab^ (47.2, 89.7)	62.6^b^ (41.9, 89.6)	73.6^b^ (61.7, 92.3)	96.4^a^ (87.0, 154)	0.94	0.03	0.01
LOX12-15 products	46.2^bc^ (30.3, 48.9)	37.4^c^ (24.9, 58.4)	44.9^b^ (38.0, 62.5)	57.1^a^ (54.7, 72.2)	0.85	0.02	0.02
LOX5 products	26.7^ab^ (18.3, 37.3)	23.9^b^ (16.0, 28.7)	25.3^b^ (21.4, 30.7)	37.1^a^ (28.7, 64.8)	0.92	0.07	0.007
CYP epoxides	1.97 (1.41, 2.19)	1.57 (1.07, 1.95)	1.83 (1.34, 2.17)	1.56 (1.27, 1.97)	0.37	0.46	0.58
Hydroxylated CYP epoxides	10.0 (6.94, 10.4)	9.19 (7.50, 10.8)	9.22 (7.29, 12.0)	9.05 (7.96, 9.63)	0.89	0.91	0.94
**sEH product to substrate ratios**							
10,11-DiHDPA/10,11-EpDPA	0.79 (0.64, 0.94)	0.79 (0.60, 0.96)	0.79 (0.57, 1.04)	0.78 (0.58, 0.93)	0.89	0.88	0.96
14,15-DiHET/14,15-EET	2.83 (2.39, 5.27)	3.31 (2.54, 4.39)	2.79 (2.31, 4.17)	2.96 (2.51, 5.95)	0.99	0.66	0.71
19,20-DiHDPA/19,20-EpDPA	2.85^ab^ (1.61, 3.11)	3.83^a^ (2.13, 4.39)	2.57^b^ (1.70, 3.31)	3.41^ab^ (2.69, 4.86)	0.22	0.25	0.23

*The superscripts denote whether averages of specific groups in a row differed at P ≤ 0.05 using Kruskal–Wallis test. If none of the groups differed from each other, no superscripts are shown. If two groups differed from each other, the significantly higher group got a superscript a, and the significantly lower group got a superscript b, and the group that did not significantly differ from the higher or the lower group got a superscript ab. If three groups differed from each other, the significantly highest group got a superscript a, the significantly lower group got a superscript b, and the group that was significantly lower than the group with a superscript b, got a, c*.

*CABG, coronary artery bypass grafting; HODE, hydroxy-octadecadienoic acid; HETE, hydroxy-eicosatetraenoic acid; EET, epoxy-eicosatrienoic acid; DiHET, dihydroxy-eicosatrienoic acid; EpDPA, epoxy-docosapentaenoic acid; DiHDPA, dihydroxy-docosapentaenoic acid; LOX, lipoxygenase; CYP, cytochrome P450; sEH, soluble epoxide hydrolases*.

### Predictive Efficacy of Oxylipins

**Figure 4:**
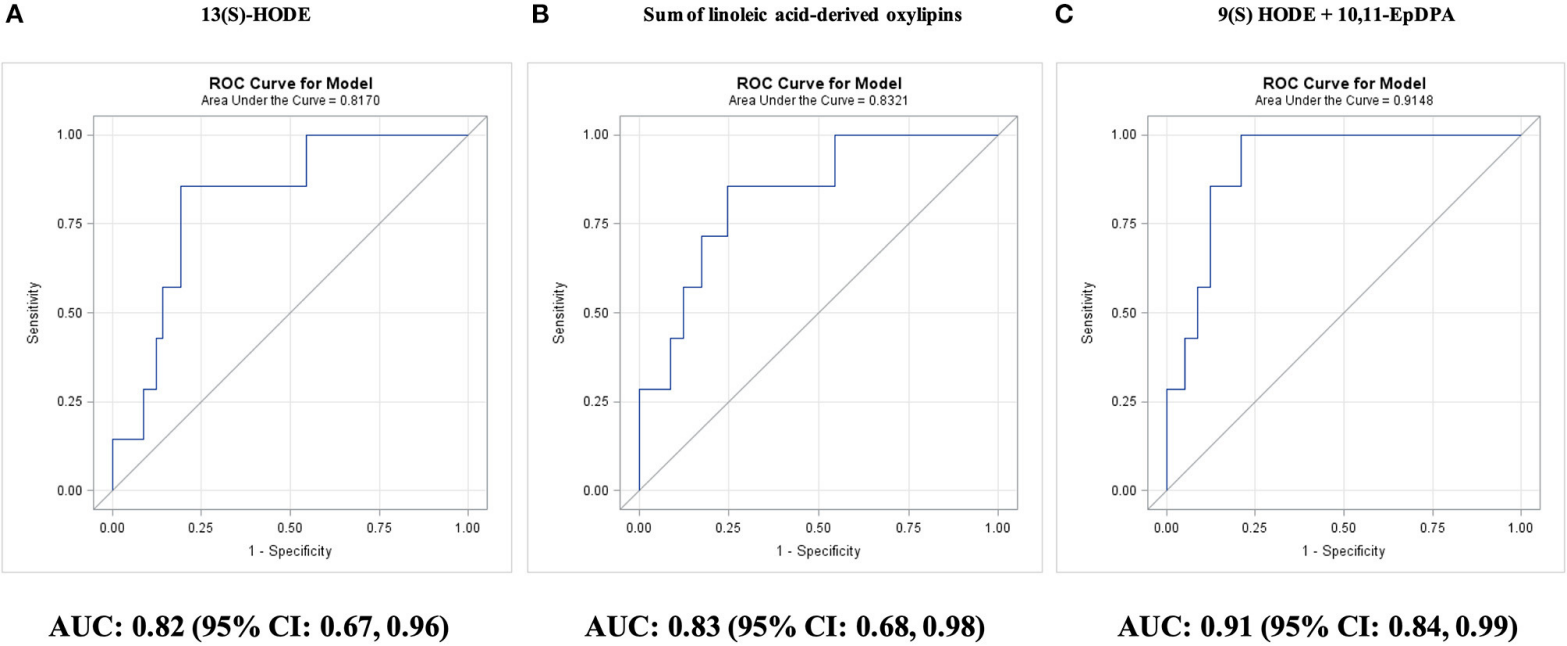
Prediction of survival during 5-year follow-up in adults with diseased coronary arteries (≥70% stenosis; *n* = 64), as shown by receiver operating characteristic (ROC) curves: **(A)** best single oxylipin model; **(B)** best single oxylipin group model; and **(C)** smallest oxylipin panel model achieving AUC ≥ 0.90.

Among individual oxylipins, survival was predicted best by LA-derived 13(S)-HODE (AUC: 0.82; 95% CI: 0.67–0.96; *P* < 0.0001); concentrations of 13(S)-HODE >42.5 nM predicted mortality in 86% nonsurviving adults with CAD and predicted survival in 81% surviving adults with CAD and 91% Astoria cohort adults (Figure 4A).

Adding 10,11-EpDPA concentrations <0.20 nM for classification, improved survival prediction to 91% surviving adults with CAD and 96% Astoria cohort adults (AUC: 0.90; 95% CI: 0.81–0.99; *P* < 0.0001). The two-oxylipin panel improved (*P* = 0.02) survival prediction compared with the 10-year Framingham general CVD risk score (AUC: 0.49; 95% CI: 0.16–0.83; *P* = 0.97).

The four remaining individual oxylipins that could significantly predict survival were ordered by *P*-value: EPA-derived 9(S)-HODE (AUC: 0.79; 95% CI: 0.62–0.96; *P* = 0.0007), ARA-derived 5-HETE (AUC: 0.73; 95% CI: 0.58–0.89; *P* = 0.01), EPA-derived 8-iso PGF3α (AUC: 0.72; 95% CI: 0.54–0.89; *P* = 0.02), and ARA-derived thromboxane B2 (AUC: 0.72; 95% CI: 0.54–0.89; *P* = 0.03). The best single predictor for survival was the sum of LA-derived oxylipins (AUC: 0.83; 95% CI: 0.68–0.98; *P* < 0.0001; Figure 4B). The targeted AUC value of at least 0.90 was achieved with a two-oxylipin panel of 9(S)-HODE and 10,11-EpDPA (AUC: 0.91; 95% CI: 0.84–0.99; *P* < 0.0001; Figure 4C).

**Figure 5:**
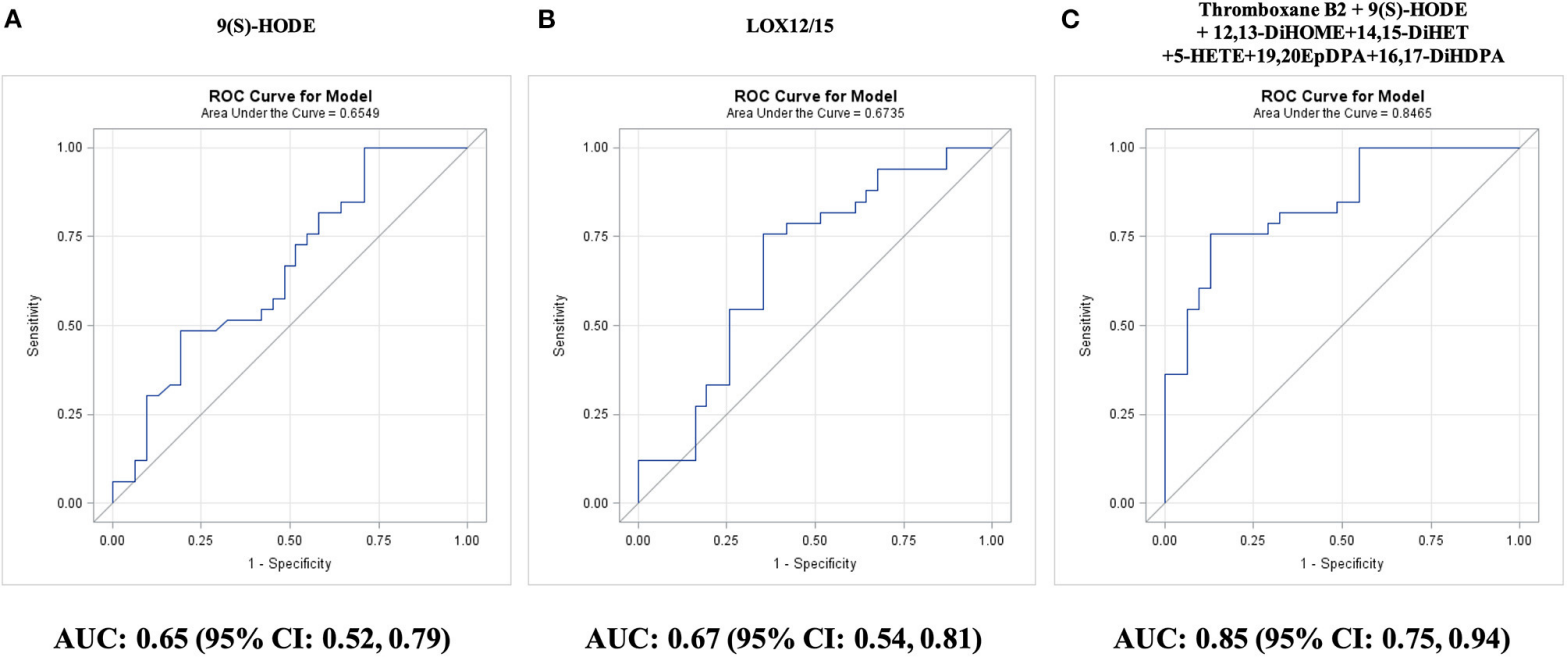
Prediction of survival without coronary artery bypass graft (CABG) surgery during 5-year follow-up in adults with diseased coronary arteries (≥70% stenosis; *n* = 64), as shown by receiver operating characteristic (ROC) curves: **(A)** best single oxylipin model; **(B)** best single oxylipin group model; and **(C)** smallest oxylipin panel model achieving AUC ≥ 0.85.

Among individual oxylipins, survival without requiring CABG was best predicted by LA-derived 9(S)-HODE (AUC: 0.65; 95% CI: 0.52–0.79; *P* = 0.03; Figure 5A). The two-remaining individual oxylipins that could significantly predict survival without requiring CABG were ordered by *P*-value: ARA-derived 15-HETE (AUC: 0.65; 95% CI: 0.52–0.79; *P* = 0.03) and ARA-derived thromboxane B2 (AUC: 0.65; 95% CI: 0.51–0.79; *P* = 0.03). The best single predictor was the sum of LOX12/15-epoxygenated oxylipins (AUC: 0.67; 95% CI: 0.54–0.81; *P* = 0.01; Figure 5B). The targeted AUC value of ≥0.85 was achieved with a linear combination of 9(S)-HODE, 5-HETE, 14,15-DiHET, thromboxane B2, 19,20-EPDPA, and 16,17-DiHDPA (AUC: 0.85; 95% CI: 0.75–0.94; *P* = 0.0001; Figure 5C). The oxylipin panel improved predictive efficacy (*P* = 0.004) compared with the 10-year Framingham general CVD risk score (AUC: 0.55; 95% CI: 0.40–0.71; *P* = 0.51).

The only single oxylipin that could significantly predict no events in CAD adults was ARA-derived 5-HETE (AUC: 0.71; 95% CI: 0.52–0.91; *P* = 0.03). In general, patients without follow-up events had oxylipin values similar to patients who died during follow-up or had a surgery for a full blockage. The 10-year Framingham general CVD risk score had an AUC of 0.62 (95% CI: 0.38–0.87; *P* = 0.33).

## Discussion

In the current study, we provide evidence that in adults with diseased coronary arteries (>70% stenosis), plasma oxylipin panels may discriminate among the number of diseased coronary arteries and predict median 5-year outcomes, which, to our knowledge, has not been previously reported.

### Analysis of Plasma Oxylipins

Novel analytical methods for extraction, detection, and data processing allow for the separation of a large number of diverse oxylipins in a short period of time (3, 12, 22, 23). In the present study, we detected and verified with standards 39 oxylipins of diverse origin and biosynthetic pathways in a 22-min LC-MS/MS run. Similar to inflammatory cytokines, low abundance, limited dynamic range, limited tissue specificity, very short half-life, significant daily fluctuation, and high inter- and intra-assay variation, limit the use of oxylipins as diagnostic biomarkers (16). For diagnostic and prognostic research, a good biomarker must have a large dynamic range within the population. In the current study, 22 oxylipins had concentrations in the linear quantification range in at least 98% of sampled adults, which allowed us to evaluate the most abundant enzymatic oxylipin pathways; however, excluded pathways generated by COX or aspirin and ROS.

### Diagnostic and Prognostic Efficacy of Oxylipins in CAD

Currently used risk assessment scores of CAD, such as the 10-year Framingham general CVD risk score, have been developed for the general population and have shown limited efficacy in high risk CAD adult management (4). In adults with significant diseased coronary arteries, a five-oxylipin panel diagnosed three diseased arteries with 100% sensitivity and 70% specificity. During a median 5-year survival, a panel of two oxylipins predicted survival with 86% sensitivity and 91% specificity. The oxylipin panels improved three diseased artery diagnosis and survival prognosis compared with the 10-year Framingham general CVD risk score.

### Clinical Relevance of Oxylipins in CAD

**Figure 6:**
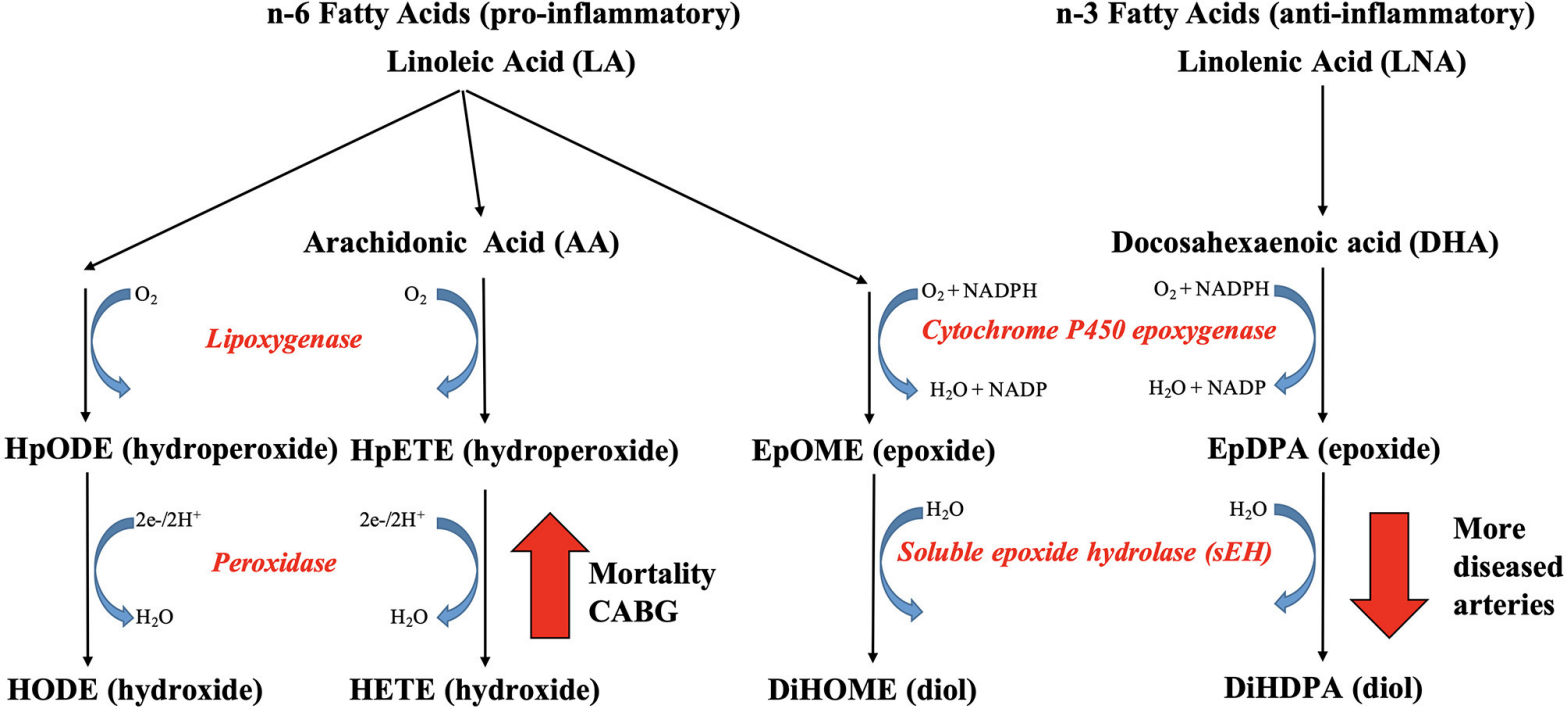
The link between plasma oxylipins and coronary artery disease. Adults with more diseased coronary arteries (≥70% stenosis) had lower plasma concentrations of hydroxylated omega-3 fatty acid-derived epoxygenated oxylipins, which was linked to decreased soluble epoxide hydrolase (sEH) activity. Nonsurviving adults with diseased coronary arteries had higher plasma concentration of oxygenated omega-6 fatty acids, which was linked to increased lipoxygenase or CYP1B1 activity.

Coronary artery disease (CAD) limits nutrient and oxygen supply to generate sufficient energy in cardiomyocytes, which becomes an even bigger challenge as the number and severity of diseased coronary arteries increase or plaques rupture with subsequent thrombus formation (5). In the present study, adults with more diseased coronary arteries (≥70% stenosis) had lower plasma concentrations of hydroxylated omega-3 PUFA-derived epoxides, specifically we observed lower levels of 19,20-DiHDPA (Figure 6). To our knowledge, the link between oxylipin concentrations and number of diseased coronary arteries has not been previously reported. The enzyme responsible for hydroxylation of epoxides is sEH, which is induced by hypoxia and has been proposed as potential pharmacological target for CAD (26–28). However, we cannot exclude the possibility that participants with CAD were already on medication that inhibited soluble CYP450 epoxide hydrolase. However, the gradual decrease in concentrations of LA-derived 12,13-DiHOME and DHA-derived DiHDPA with increasing diseased artery number support the hypothesis that the lower concentrations are a response to the hypoxia caused by arterial occlusions (29).

Five-year survival and no CABG surgery was linked to lower concentrations of oxygenated omega-6 PUFA LA and ARA, specifically lower concentrations of LA-derived mid-chain HODE and ARA-derived mid-chain HETE, which are either generated by oxygenation of lipoxygenases or hydroxylation of CYP1B1 (Figure 6). In support, elevated 15-HETE concentrations and LOX-15 enzymatic activity have been reported in ischemic heart disease and hypoxic human cardiomyocytes and cardiac endothelial cells (15), supporting our hypothesis that the elevated mid-chain HETE and HODE concentrations are a response to the hypoxia caused by the arterial occlusions. High concentrations of HETE, including 5-HETE, 12-HETE, and 15-HETE, were reported in atherosclerotic plaques, especially in those that were more likely to rupture (14). Elevated circulating concentrations of 5-HETE and 12-HETE were observed in individuals after cardiac surgery (16). Elevated concentrations of 5-HETE, 12-HETE, and 15-HETE were reported in individuals with acute cardiac syndrome (17). Elevated circulating concentrations of 5-HETE, 12-HETE, and 15-HETE were reported in individuals with CAD by Xu et al. (20), whereas only numerical increases were reported by Shishebor et al. (19) and Auguet et al. (21); the latter did not quantify 5-HETE. The role of elevated mid-chain HETE in cardiovascular dysfunction has been well documented, whereas less is known of the role of mid-chain HODE (3, 30, 31). Inhibition of the oxygenation step of the LOX pathway has been proposed as treatment option for CAD management (30), suggesting clinical relevance of the identified oxylipins as indicator of chronic hypoxia.

### Limitations of the Study

First, the number of adults with CAD were relatively small and came from a high-risk group, which underwent coronary angiography, but in whom the presence and extent of CAD was clearly defined. Second, all but five adults with CAD were on medical therapy for treatment for hypertension, hyperlipidemia, and/or diabetes, which could have influenced the levels of oxylipins. Third, differences in collateral blood flow may have impacted oxylipin concentration, which was not assessed in this cohort. Fourth, most adults with CAD had to undergo revascularization shortly after angiography, which may impact later outcomes. Fifth, follow-up time was limited to <6 years, which impacted the number of outcomes. Sixth, diagnostic and prognostic oxylipin panels could not be validated due to the limited clearly defined population size.

## Summary and Conclusion

In summary, we observed a link between plasma oxylipin concentrations and CAD severity. Concentrations of six oxylipins decreased with the number of diseased arteries; a panel of five oxylipins diagnosed three diseased arteries with 100% sensitivity and 70% specificity. Concentrations of five oxylipins were lower and one oxylipin was higher with survival; a panel of two oxylipins predicted survival during follow-up with 86% sensitivity and 91% specificity. Plasma oxylipins may assist in diagnosis and prognosis of CAD in high-risk adults in combination with standard risk assessment tools. Our promising results require confirmation in larger unselected populations.

## Data Availability Statement

The original contributions presented in the study are included in the article/Supplementary Material, further inquiries can be directed to the corresponding author/s.

## Ethics Statement

This study was approved by the Institutional Review Board of the Oregon Health and Science University (OHSU) in Portland, Oregon. The patients/participants provided their written informed consent to participate in this study.

## Author Contributions

DL, MG-J, GB, AA, DR, NA, CM, and SK: conceptualization. DL, MG-J, GB, AA, AV, JM, DJ, and CM: data curation. DL, MG-J, and GB: formal analysis and writing (original draft). MG-J, DJ, CM, and SK: funding acquisition. DL, MG-J, GB, AA, AV, JM, DJ, DR, NA, CM, and SK: investigation and writing (review and editing). DL, MG-J, GB, AA, DJ, CM, and SK: methodology. DL, MG-J, NA, CM, and SK: project administration. MG-J and GB: software and visualization. GB, DJ, CM, and SK: supervision. DL, MG-J, GB, DJ, CM, and SK: validation. All authors contributed to the article and approved the submitted version.

## Conflict of Interest

The authors declare that the research was conducted in the absence of any commercial or financial relationships that could be construed as a potential conflict of interest.

## Abbreviations

ACN: acetonitrile
ARA: arachidonic acid
CAD: coronary artery disease
COX: cyclooxygenases
CUDA, 1-cyclohexyl ureido: 3-dodecanoic acid
CVD: cardiovascular diseases
CYP450: cytochrome P450
CYPEPOX: CYP epoxide
DHA: docosahexaenoic acid
EPA: eicosapentaenoic acid
FA: fatty acid
4-HNE: 4-hydroxynonenal
HPLC-MS/MS: high-performance liquid chromatography tandem mass spectrometry
IMS: ionization mass spectrometry
IPA: isopropanol
LA: linoleic acid
LDL: low-density lipoproteins
LNA: linolenic acid
LOD: limit of detection
LOQ: limit of quantification
LOX: lipoxygenases
MDA: malondialdehyde
MRM: multi-reaction monitoring
PUFA: polyunsaturated fatty acids
ROS: reactive oxygen species
RT: retention time
sEH: soluble epoxide hydrolases.

## Appendix

8-iso PGF3a, 9S,11R,15S-trihydroxy-5Z,13E,17Z-prostatrienoate;

thromboxane B2, 9S,11,15S-trihydroxy-thromboxa-5Z;

17,18-DiHETE, 17,18-dihydroxy-5Z,8Z,11Z,14Z-eicosatetraenoic acid;

leukotriene B4, (5S,6Z,8E,10E,12R,14Z)-5,12-dihydroxyicosa-6,8,10,14-tetraenoic acid;

11,12-DiHETE, 11,12-dihydroxy-5Z,8Z,14Z,17Z-eicosatetraenoic acid;

12,13-DiHOME, 12,13-dihydroxy-9Z-octadecenoic acid;

5,6-DiHETE, 5,6-dihydroxy-8Z,11Z,14Z,17Z-eicosatetraenoic acid;

19,20-DiHDPA, 19,20-dihydroxy-4Z,7Z,10Z,13Z,16Z-docosapentaenoic acid;

14,15-DiHET or 14,15-DiHETrE, 14,15-dihydroxy-5Z,8Z,11Z-eicosatrienoic acid;

16,17-DiHDPA, 16,17-dihydroxy-4Z,7Z,10Z,13Z,19Z-docosapentaenoic acid;

13,14-DiHDPA, 13,14-dihydroxy-4Z,7Z,10Z,16Z,19Z-docosapentaenoic acid;

10,11-DiHDPA, 10,11-dihydroxy-4Z,7Z,13Z,16Z,19Z-docosapentaenoic acid;

7,8-DiHDPA, 7,8-dihydroxy-4Z,10Z,13Z,16Z,19Z-docosapentaenoic acid;

20-HETE, 20-hydroxy-5Z,8Z,11Z,14Z-eicosatetraenoic acid;

13(S)-HODE, 13S-hydroxy-9Z,11E-octadecadienoic acid;

9(S)-HODE, 9S-hydroxy-10E,12Z-octadecadienoic acid;

15-HETE, 15-hydroxy-5Z,8Z,11Z,13E-eicosatetraenoic acid;

17,18-EpETE, 17,18-epoxy-5Z,8Z,11Z,14Z-eicosatetraenoic acid;

12-HETE, 12-hydroxy-5Z,8Z,10E,14Z-eicosatetraenoic acid;

5-HETE, 5-hydroxy-6E,8Z,11Z,14Z-eicosatetraenoic acid;

19,20-EpDPA or 19,20-EpDPE, 19,20-epoxy-4Z,7Z,10Z,13Z,16Z-docosapentaenoic acid;

14,15-EET or 14,15-EpETrE, 14(15)-epoxy-5Z,8Z,11Z-eicosatrienoic acid;

10,11-EpDPA or 10,11-EpDPE, (4Z,7Z)-9-[3-(2Z,5Z,8Z)-2,5,8-undecatrien-1-yl-2-oxiranyl]-4,7-nonadienoic acid;

11,12-EET or 11,12-EpETrE, 11(12)-epoxy-5Z,8Z,14Z-eicosatrienoic acid.
