# Supplementary material for: Plasma Oxylipins: A Potential Risk Assessment Tool in Atherosclerotic Coronary Artery Disease
Source: Front Cardiovasc Med. 2021 Apr 21;8:645786. doi: 10.3389/fcvm.2021.645786 (PMC8097092; doi:10.3389/fcvm.2021.645786)
Supplement: Supplementary file 1 [file Data_Sheet_1.docx]

**Figure S1.** Extracted ion chromatogram of the 60 oxylipins (20 of them deuterated) contained in our *in-house* library. Each color represents a different transition.
