# Supplementary material for: Plasma Oxylipins: A Potential Risk Assessment Tool in Atherosclerotic Coronary Artery Disease
Source: Front Cardiovasc Med. 2021 Apr 21;8:645786. doi: 10.3389/fcvm.2021.645786 (PMC8097092; doi:10.3389/fcvm.2021.645786)
Supplement: Supplementary file 2 [file Data_Sheet_2.docx]

**Table S1.** Detailed list of multi-reaction monitoring (MRM) transitions for the deuterated-oxylipins (surrogates) and CUDA (12-[[(cyclohexylamino) carbonyl] amino]-dodecanoic acid) used as internal standards for our analysis. Compounds are ordered based on retention time (RT).

RT: retention time (min); DP: declustering potential (V); CE: collision energy (V); S/N: Signal to Noise Ratio; LOD: limit of detection; LOQ: limit of quantification.
