# Supplementary material for: Plasma Oxylipins: A Potential Risk Assessment Tool in Atherosclerotic Coronary Artery Disease
Source: Front Cardiovasc Med. 2021 Apr 21;8:645786. doi: 10.3389/fcvm.2021.645786 (PMC8097092; doi:10.3389/fcvm.2021.645786)
Supplement: Supplementary file 3 [file Data_Sheet_3.docx]

**Table S2.** Detailed list of multi-reaction monitoring (MRM) transitions for the oxylipins contained in our *in-house* library. Compounds are ordered based on retention time (RT).

RT: retention time (min); DP: declustering potential (V); CE: collision energy (V); S/N: Signal to Noise Ratio; LOD: limit of detection; LOQ: limit of quantification.
